# The impact of the COVID-19 pandemic on the development of acute and transient psychotic disorders

**DOI:** 10.1192/j.eurpsy.2021.693

**Published:** 2021-08-13

**Authors:** A. Pérez-Balaguer, L.M. Solari-Heresmann, E. Gil-Benito, B. Sanz-Aranguez, L. Gayubo-Moreo

**Affiliations:** Psychiatry, Hospital Universitario Puerta de Hierro de Majadahonda, Madrid, Spain

**Keywords:** COVID-19, pandemic, Acute and transient psychotic disorder, Reactive psychosis

## Abstract

**Introduction:**

Since the declaration of the COVID-19 pandemic, several studies have demonstrated its considerable psychological impact. The isolation and social distancing, the increased fear of being infected or infecting others and the insecurity generated by the economic impact, could contribute to an increase in the incidence of mental health issues, such as psychotic disorders.

**Objectives:**

The aim is to discuss four clinical cases in order to provide further evidence on this matter.

**Methods:**

We report on three females and one male with no personal psychiatric history who were admitted to a tertiary hospital during the first three months after the declaration of the pandemic. The average age was 44,25 ± 14,97 years.

**Results:**

All patients met the International Statistical Classification of Diseases (ICD-10) criteria for acute and transient psychotic disorder. All of the episodes were triggered by the stress generated from the COVID-19. Complementary tests were unremarkable. They all tested negative for SARS-CoV-2. Rapid discharge with favorable response to relatively low doses of antipsychotics was possible with a mean length of stay of 7,25 ± 2,86 days. In two of the cases the delirious content was predominantly marked by the coronavirus itself.

**Conclusions:**

It has been suggested that the intense psychosocial stress associated with a new life-threatening disease and national lockdown restrictions could be triggers for new-onset psychotic disorders. Some authors have reported cases similar to ours, which means that we could be experiencing and increase in the incidence of psychotic disorders due to the exceptional circumstances we are living around the world.

